# Implication of red meat consumption habits in serum uric acid levels and mood disorders among first-trimester pregnant women

**DOI:** 10.1186/s40795-023-00769-y

**Published:** 2023-09-29

**Authors:** Mudi H. Alharbi, Nora H.J. Alharbi, Ibtihal A. Brnawi, Elham H. Atiq

**Affiliations:** 1 Clinical Nutrition Department, College of Applied Medical Sciences, Taibah University, Madinah, 42353 Saudi Arabia; 2 Department of Pharmacology and Toxicology, College of Pharmacy, University of Ha’il, Ha’il, 81442 Saudi Arabia; 3 Clinical Nutrition Department, Psychiatric Specialist Hospital, King Salman Medical City, Madinah, Saudi Arabia

**Keywords:** Anxiety, Depression, Meat consumption, Pregnant women, Uric acid

## Abstract

**Background:**

Dietary pattern involving meat consumption has an association with serum uric acid level which subsequently has an impact on moods. However, this relationship is not clearly established in pregnant women, particularly those who are accustomed to daily meat consumption.

**Objective:**

This study investigated the relationship between red meat consumption and uric acid level and the subsequent impact on mood disorders in 1st trimester pregnant women.

**Methodology:**

A total of 92 pregnant women in their first trimester (8–12 weeks), were selected for this study. Socio-demographic characteristics including age, body mass index (BMI), educational qualification, sleep hours, blood pressure and exercise status were recorded. To assess meat consumption, classification based on the recruited population consumption was divided into low and high meat consumption groups. Serum uric acid level was estimated in plasma. Mood disorder, namely, depression and anxiety were assessed using a self-reported Hospital Anxiety and Depression Scale (HADS) questionnaire. Collected data was analysed using different statistical tools.

**Results:**

Logistic regression analysis showed higher odds of depression (OR = 0.059, 95% CI 0.02–0.172, p < 0.001) and anxiety (OR = 0.144, 95% CI 0.055–0.375, p < 0.001) in the high meat consumption group. Further, the potential confounders, high BMI and less exercise increased the odds of depression and anxiety in high meat consumption groups. Linear regression analysis revealed a significant influence of meat consumption on uric acid level (*F* (1, 90) = 305.385, p < 0.01).

**Conclusions:**

The study recommends regular clinical screening of mood disorders, and recommends reasonable consumption of lean meat and/or replacing some portions with fish, as well as, a commitment to eating a healthy, balanced diet. It also suggests extensive studies because it could be linked to postpartum mood disorders among those who consume red meat every day.

**Supplementary Information:**

The online version contains supplementary material available at 10.1186/s40795-023-00769-y.

## Introduction

One of the nations in the world with the highest per capita meat consumption is Saudi Arabia. In 2024, it was expected that Saudi Arabians will consume more than 2.1 million tons of meat [1). The Department of Health and Social Care recommends no more than 70 gm of meat per day [2], although the average yearly intake of red meat per person in Saudi Arabia is 50 kg [3], or approximately 138 gm, per person. Furthermore, Saudi Arabia consumes more poultry than any other country in the world, with an average yearly per-person consumption of 41.6 kg of chicken as opposed to 12.5 kg globally [4]. Consuming a lot of meat is linked to a number of illnesses and risk factors, many of which are exacerbated by pregnancy and have an impact on the fetus [5]. One of them is hyperuricaemia or increased serum uric acid level is associated with multiple pathological symptoms including hypertension, kidney disease and gout [6]. Multitude of factors including diet pattern and hereditary contributes to the risk of increased uric acid in the serum. For over centuries, meat has been an important dietary source of proteins, vitamins and minerals. Yet meat products high in purine such as red meat or organ meats are associated with increased serum uric acid level [6, 7]. Purines naturally present in the food is metabolised through purine metabolism to uric acid which exerts neuroprotective effect due to their antioxidant property [8]. Uric acid is produced by a xanthine oxidoreductase (XOR)-catalysed process, which has been demonstrated to operate as both an antioxidant and a pro-oxidant by producing and regulating reactive oxygen species (ROS) [9]. The significance of uric acid, particularly in the regulation of oxidative stress (i.e., ROS), is currently being debated. According to Kurajoh et al., uric acid increases oxidative stress, particularly in females, generating an imbalance between XOR-independent ROS generation and scavenging [10]. Besides that uric acid is also associated with certain pathophysiological conditions such as gout [11] and mental disorders, namely, depression and anxiety [12] The reference range for uric acid is age and sex specific. In adult pre-menopausal females, uric acid level in the range 2.6–5.7 mg/dl is considered normal and uric acid level above 7.0 mg/dl is defined as hyperuricemia [6]. However, in pregnant women throughout their gestation, a fluctuation in the uric acid level is noted. In the first trimester, oestrogen’s uricosuric effect reduces the uric acid level below to 3 mg/dl, however, during the third trimester the level increases to 4–5 mg/dl [13]. Thus, the prognostic value of uric acid in the determination of preeclampsia and gestational hypertension has frequented the clinical utility of uric acid measurement in pregnant women [14].

According to studies, the dietary pattern of pregnant women has a larger implication on maternal and child outcomes [15]. Thus, these factors could contribute to mood disorders and lead to adverse maternal outcomes such as postpartum depression and child outcomes like shorter gestation and poor development [16]. Hence, the clinical management of mood disorders during pregnancy has gained a lot of attention. Research evidence indicates association of meat consumption with physiological health. Meat consumption in particular the red meat including beef, pork and veal was associated with higher risk of depression or anxiety [17–19]. Further, another biochemical parameter such as serum uric acid has been proposed to be used as a marker for different subtypes of depression disorder, namely, bipolar disorder depressive episode and major depressive disorder [12, 20]. Thus, a link between meat consumption, uric acid and mood disorder can be anticipated. In the literature, high meat consumption is linked to increased uric acid [21] and higher serum uric acid level is associated with depression in the general population [22]. However, a similar association in pregnant women has not been explored. Based on literature findings, an evaluation of diet throughout the gestation could be a potential determinant of outcomes during pregnancy. This study conducted the evaluation pattern of meat consumption, serum uric acid levels and mood disorders in women with less than 12 gestational weeks.

## Methodology

### Sample population

A cross-sectional study in the cities of Riyadh and Madinah between November 2021 and ending in September 2022, surveyed 92 pregnant women in their first trimester. Pregnant women not exceeding 12 weeks of gestation were included in the study. Pregnant women beyond the gestational age of 12 weeks and with incidences of gestational diabetes mellitus, gout, endocrine disorders and chronic renal diseases were excluded from the study. In addition, pregnant women who consumed medication such as aspirin, caffeine, diuretics and phenothiazines which are known to increase uric acid level in the blood were excluded from the study. Participants’ sociodemographic information, including their age, education, average daily sleep time, amount of exercise, and BMI, was collected. In order to determine if individuals were eligible, questions concerning health and lifestyle were asked as part of the questionnaire.

### Assessment of the meat intake, and other metrics

For those who met the study criteria, the study protocols were described to them, as well as, a link to complete the questionnaire was sent to them. Food consumption information was gathered from a 24-hour dietary recall for 3 days within a week and the average was calculated (by asking the participants about their recent meals during 24 h and including size illustrations to help participants estimate their intake) and a meat frequency questionnaire (mFQ), which was based on the technique’s of the Food Frequency Questionnaire (FFQ) and was tailored to Saudi culture or meat consumption customs and was in Arabic language. The study instrument’s face validity among the Saudi population was validated by delivering the questionnaire to the seven professionals who were chosen randomly [23]. Also, a prior study (unpublished) looked at the validity of the meat questionnaire. Cronbach’s alpha was calculated to be (0.89). As a result, the questionnaire’s internal dependability and reliability is rated as extremely high. On average, every individual consumes 75 gm of meat per day, which has been collected by a 24-hr dietary recall which was performed by trained professional dietitians. The most frequency of meat consumption (Table 1). For the analysis purpose, total meat consumption was divided into low meat and high meat based on the percentile value. The estimated 50th percentile value for total meat consumption was 16. Therefore, values ≤ 16 were taken as low meat and > 16 was taken as high. For the blood pressure BP, height and weight and serum uric acid measurements, the participants were requested to check in the week when the 24-hour was collected, and that was in specific clinics which used the same measurement units. The BMI was calculated by researchers and classified according to the Institute of Medicine (IOM), which has established weight gain recommendations based on pre-pregnancy BMI. These guidelines are for BMI 19.8 kg/m2 total weight gain of 12.5 to 18 kg, BMI = 19.8 to 26.0 kg/m2 total weight gain of 11.5 to 16 kg, BMI > 26.0 to 29.0 kg/m2 total weight gain of 7.0 kg, and BMI > 29.0 kg/m2 total weight gain of 7.0 kg [24]. However, all the costs associated with this were covered by the researchers.

**Table 1:** Frequency of meat consumption

	Never or less than once a month	1 to 3 times a month	Once a week	2 to 4 times a week	5 to 6 times a week	Once a day
Beef burger	37 (40.22)	25 (27.17)	10 (10.87)	14 (15.22)	6 (6.52)	0 (0)
Red meat	17 (18.48)	24 (26.09)	19 (20.65)	23 (25)	7 (7.61)	2 (2.17)
Sausage meat, pepperoni, mortadella	31 (33.7)	21 (22.83)	12 (13.04)	12 (13.04)	14 (15.22)	2 (2.17)
Liver, kidney, heart and lung	29 (31.52)	17 (18.48)	5 (5.43)	19 (20.65)	19 (20.65)	3 (3.26)

### Mood questionnaire

The participants were asked to fill the questionnaire for the assessment of mood disorders. The mood status related to depression and anxiety was assessed using a commonly used self-assessment HADS questionnaire. HADS is a common used self-assessment scale developed to evaluate psychological disorder in non-psychological patients and validated in Arabic version [25]. The HADS contains 14 items and has two subscales – depression (7 items) and anxiety (7 items). Each item was scored on a four-point scale and scores from 0 to 7 was considered as normal, score of 8 to 10 represented borderline depression/anxiety and score of 11 to 21 represented abnormal or severe depression/anxiety.

### Statistical analysis

All tests were performed using SPSS v24.0. The participant’s baseline characteristics were tabulated in the form of frequency and percentage. Linear regression was performed to analyse the relationship between meat consumption and uric acid. Further, logistic regression analysis was performed to examine the impact of meat consumption, BMI and exercise on anxiety and depression. The variance accounted for in the dependent variable (anxiety and depression) due to meat consumption and serum uric acid level was quantified by calculating Cox and Snell, and Nagelkerke pseudo R^2^ values. A p-value less than 0.05 was considered statistically significant.

## Results

### Baseline characteristics

Table 2 shows the baseline characteristics of 92 pregnant patients who were enrolled in the present study. The selected population were concentrated in the age category of 30–39 years (56.52%) and were graduates (78.2%). Most of the participants slept 6 h a day (47.83%), had a high BMI (53.2%) and performed some kind of exercise (52.17%). Most of the participants had borderline depression and abnormal depression (43.48% and 14.13%) and anxiety (47.83% and 18.48%). Based on total meat consumption, about 43.48% had high meat consumption. The mean value of SBP was 122.31 ± 3.87 mm Hg, DBP was 83.85 ± 6.65 mmHg and estimated serum uric acid level was 3.544 ± 0.91 mg/dl in the sample population.

**Table 2:** Baseline characteristics

	Frequency (%)
Age	
19–29	38 (41.3)
30–39	52 (56.52)
40–45	2 (2.17)
**Educational level**	
High school degree	18 (19.57)
Graduation	72 (78.26)
Post-graduation	2 (2.17)
**Average sleeping hours/day**	
Less than 6 h a day	11 (11.96)
6 h a day	44 (47.83)
More than 6 h a day	37 (40.22)
**Exercise (walking)**	
Yes	48 (52.17)
No	44 (47.83)
**BMI**	
Normal	43 (46.74)
Over weight	24 (26.09)
Obesity	25 (27.17)
**Anxiety**	
Normal	31 (33.7)
Borderline	44 (47.83)
Abnormal	17 (18.48)
**Depression**	
Normal	39 (42.39)
Borderline	40 (43.48)
Abnormal	13 (14.13)
Meat consumption*	
Low meat	52 (56.52)
High meat	40 (43.48)

* p < 0.05

Most pregnant participants consumed beef burger (27.17%), red meat (26.09%) and sausage meat, pepperoni and mortadella (22.83%) 1 to 3 times a month. Consumption of meat comprising of liver, kidney, heart and lung was more frequent from 2 to 6 times per week (41.2%).

### Impact of meat consumption, exercise and BMI on depression

A logistic regression was conducted with meat consumption (low meat as reference) as independent variable, exercise (yes to exercise as reference) and BMI (normal as reference) as confounding variable, and depression (no depression as reference) as independent variable (Table 3). The model 1 indicates higher odds of depression in the high meat consumption group than the low meat consumption group (OR = 0.059, 95% CI 0.02–0.172, p < 0.001). Model 2 and model 3 involve confounding factors, namely, BMI and exercise to predict depression. The odds of depression were higher in high meat consumption with no exercise than high meat consumption with exercise (OR = 0.053, 95% CI 0.015–0.187, p < 0.001) (model 2). In terms of BMI classification, the high meat consumption group with higher BMI, representing overweight, showed increased odds of depression than the high meat consumption group with normal BMI (OR = 0.040, 95% CI 0.007–0.221, p < 0.001) (model 3). Further, Omnibus Tests of Model Coefficients indicated that all models predicted depression – model 1 (χ2 = 35.196, p < 0.01), model 2 (χ2 = 60.83, p < 0.01) and model 3 (χ2 = 78.61, p < 0.01) (Data not shown).

**Table 3:** Impact of meat consumption, exercise and BMI on depression

		B	p value	OR (95% CI)
Model 1	**Meat consumption**			
	High meat	-2.833	0.000	0.059 (0.02 − 0.172)
	Low meat	Ref		
Model 2	**Meat consumption**			
	High meat	-2.372	0.000	0.093 (0.026 − 0.337)
	Low meat	Ref		
	**Exercise**			
	No	-2.932	0.000	0.053 (0.015 − 0.187)
	Yes	Ref		
Model 3	**Meat consumption**			
	High meat	-3.026	0.000	0.049 (0.009 − 0.258)
	Low meat	Ref		
	**BMI classification**		0.000	
	Over weight	-3.217	0.000	0.040 (0.007 − 0.221)
	Obesity	-5.399	0.000	0.005 (0.000 − 0.055)
	Normal	Ref		

### Impact of meat consumption, exercise and BMI on anxiety

Additionally, another logistic regression was conducted with meat consumption (low meat as reference) as independent variable, exercise (yes to exercise as reference) and BMI (normal as reference) as confounding variable, and anxiety (no anxiety as reference) as independent variable (Table 4). The model 1 indicated higher odds of anxiety in the high meat consumption group than the low meat consumption group (OR = 0.144, 95% CI 0.055–0.375, p < 0.001) (model 1). Further, a high meat consumption group with no exercise showed increased odds of anxiety than high meat consumption with exercise (OR = 0.21, 95% CI 0.076–0.585, p < 0.001) (model 2). In terms of BMI classification, high meat consumption group with higher BMI, representing overweight (OR = 0.123, 95% CI 0.035–0.43, p < 0.001) and obesity (OR = 0.101, 95% CI 0.027–0.379, p < 0.001) showed increased odds of anxiety than high meat consumption group with normal BMI (model 3). Further, Omnibus Tests of Model Coefficients indicated that all models predicted anxiety – model 1 (χ2 = 18.14, p < 0.01), model 2 (χ2 = 27.35, p < 0.01) and model 3 (χ2 = 36.81, p < 0.01) (Data not shown).

**Table 4:** Impact of meat consumption, exercise and BMI on anxiety

		B	p value	OR (95% CI)
Model 1	**Meat consumption**			
	High meat	-1.938	0.000	0.144 (0.055 − 0.375)
	Low meat	Ref		
Model 2	**Meat consumption**			
	High meat	-1.363	0.011	0.256 (0.09 − 0.731)
	Low meat	Ref		
	**Exercise**			
	No	-1.559	0.003	0.21 (0.076 − 0.585)
	Yes	Ref		
Model 3	**Meat consumption**			
	High meat	-1.328	0.017	0.265 (0.089 − 0.786)
	Low meat	Ref		
	**BMI classification**		0.000	
	Over weight	-2.094	0.001	0.123 (0.035 − 0.43)
	Obesity	-2.298	0.001	0.101 (0.027 − 0.379)
	Normal	Ref		

### Impact of uric acid on depression and anxiety

Logistic regression analysis (Tables 5 and 6) showed that serum uric acid level significantly increased the risk of depression (OR: 0.077, 95% CI 0.03–0.198) and anxiety (OR: 0.22, 95% CI 0.111–0.436) in pregnant women who consumed meat. The pseudo-R square value of 0.427 and 0.23 (Cox & Snell), and 0.570 and 0.318 (Nagelkerke) explains variation of 42.7% and 57.0% in depression, and 23.0% and 31.8% in anxiety due to increased serum uric acid level.

**Table 5:** Impact of uric acid on depression

	B	p value	OR (95% CI)
Uric acid	-2.570	0.000	0.077 (0.03 − 0.198)
Constant	8.228	0.000	

**Table 6:** Impact of uric acid on anxiety

	B	p value	OR (95% CI)
Uric acid	-1.51	0.000	0.22 (0.111 − 0.436)
Constant	4.635	0.000	

### Impact of meat consumption on uric acid

In linear regression analysis, the adjusted R value of 0.770 indicated a strong and positive relationship between meat consumption and uric acid. Further, beta coefficient value indicated that for every one unit increase in meat consumption the serum uric acid level increased by 0.879 times (p < 0.01) (Table 7). Overall, in pregnant women, meat consumption influenced serum uric acid level.

**Table 7:** Coefficients for the impact of meat consumption on uric acid

Adjusted R Square		Unstandardized Coefficients		T	Sig.
		**B**	**Std. Error**		
0.770	Meat consumption	0.879	0.050	17.475	0.000

## Discussion

In the present study, a total of 92 women in the first trimester of pregnancy were enrolled for the study. The study outcome linked high meat consumption with increased risk of anxiety and depression in pregnant women. In literature, the relationships between meat consumption and anxiety/depression have been mixed due to factors like methodologic rigor, confidence in results and validity of interpretation. In a pool study, 3 out of 18 studies indicated that avoiding meat consumption improved psychological health as revealed by reduced anxiety or depression suggesting that high meat consumption can reduce psychological health, which supports our data [26]. Since in the present study, regular or high meat consumption was linked to purine-rich meat food like red meat/organ meat, in support of our findings Mofrad et al. [27] found that red meat consumption increased distress, depression and anxiety in women. Other evidence also supports association of meat consumption, specifically red meat consumption, with higher chances of depression in the general population [18]. Furthermore, meat consumption was associated with serum uric acid level in this study. Comparatively higher uric acid level was noted in the women population who regularly consumed meat than vegetables or fish [28]. In a population-based cohort study involving 16,670 individuals’ serum uric acid level was found to be higher in the population who consumed meat food, specifically, beef, pork or lamb. Authors showed the contribution of diet rather than genetics in the increased serum uric acid level [29]. In a cross-sectional study, a high purine diet was a major contributor to increased uric acid level [30]. Thus, it can be inferred that meat consumption, in particular the purine-rich meat products like red meat (beef, organ meat) are one of the contributing factors of increased serum uric acid level in pregnant women included in this study.

In the present study, serum uric acid level was associated with anxiety and depression in pregnant women. On the contrary, the majority of references indicate an inverse relationship between uric acid and mood disorder (anxiety and depression). Wen et al. [31] suggests low uric acid level as a characteristic of depression. Similar findings on reduced plasma uric acid level in depressive and anxiety disorder is reported [32]. Majority of studies suggest the neuroprotective effect of uric acid against depression and anxiety [33]. According to Meng et al. [12] serum uric acid levels vary across different types of disorder and therefore can be used as a marker for recurrent depressive episodes, major depressive disorder and depression with anxiety. The possible explanation to association of uric acid level to increased anxiety or depression can be explained using purinergic dysfunction, however, more detailed analysis is suggested. Researchers associate the purinergic system dysfunction to mood disorders. Under this context, Ali-Sisto et al. [34] associated purine catabolism with psychiatric disorders depression (major depressive disorder). According to their study, accumulation of one of the purine metabolites, namely, xanthine is higher in people with depression than in healthy persons, therefore, authors postulated that the increased xanthine production may be the body’s compensatory mechanism seeking to increase the production of uric acid in order to combat increased oxidative stress caused by depression. Authors did not estimate the serum uric acid level, however, a link between increased uric acid and mood disorders can be anticipated which will need more evaluation. In a similar fashion, increased uric acid level or purinergic dysfunction is also associated with bipolar disorders [35, 36] Studies link increased uric acid with bipolar disorder and has been used clinically as a biomarker to differentiate different types of disorders [37]. In concordance with the present finding, Tao and Li [22] suggested use of high serum uric acid level as a biochemical index for the early diagnosis of adolescent depression. However, limited by sample size the study was performed on male adolescent subjects. In this particular study adolescence depression was linked to DNA oxidative damage of neurons in the brain showing involvement of serotonin (5-HT). While there are several variables that might cause mood swings, the most common is a rapid increase in pregnancy hormones. During the first few days of pregnancy, a woman’s body produces a rush of oestrogen and progesterone. These two hormones can have an impact on one’s mental health. That could lead to activity of these hormones in the brain area that governs mood, based on this hormone is linked to anxiety, emotion, and depression [38, 39]. In a prospective longitudinal study of 417 pregnant women in the United Kingdom, England, 41 (9.8%) were depressive during pregnancy, and 31 (7.4%) afterwards [40]. Accordingly, we can suggest that after increasing the hormones in the pregnancy, maybe the body stabilizes or controls the hormones afterwards and mood swings become less apparent gradually. So, that cannot happen without controlling other triggers that could affect one’s mood during pregnancy, which are eating habits, especially for red meat and increased uric acid. As a result, one study discovered that untreated depression during pregnancy is a key indicator of postpartum depression [41]. However, this study did not ignore the contribution of hormones to mood swings during pregnancy, which was not measured but tried to help to discover the contributing factor that could help to control the mood swings during pregnancy and afterwards.

Besides that, confounding factors can also determine the anxiety and depression in individuals. In the present study, BMI and exercise were the potential confounders. Given that, pregnant women with high BMI and with no exercise had higher odds of depression and anxiety. In general, obese and physically less active individuals are known to have higher risk of depression [42, 43]. Less physical activity was shown to be associated with obesity and depression in pregnant women [44] which supports the present finding. Kołomańska et al. [45] states that pregnant women with regular physical activity have better psychological health. Overall, from the data it can be inferred that high meat consumption with less physical activity and obesity can increase the vulnerability of pregnant women to poor psychological health.

To summarize, the present findings associate high meat consumption with increased uric acid and mood disorder. There is no denial that animal-based food is a good source of vitamins and minerals [46], however, based on the present and literature findings moderate meat consumption or adherence to recommended meat consumption guidelines is suggested to achieve better outcomes. According to Kaneko et al. [8] moderation in the intake of purine-rich food containing high ratio of hypoxanthine including animal meats, fish, organs such as the liver and fish milt, and yeast is recommended to avoid increased serum uric acid level. This is to be noted that the recruited participants did take supplements like folic acid multivitamins and mineral complexes thus moderation of meat product will not affect the recommended level of vitamins and minerals uptake during the pregnancy. The primary limitation was small sample size. In addition, somatic symptoms like fatigue and contribution of hormonal changes to anxiety or depression during pregnancy were not accounted for. A longitudinal study with a larger sample size will strengthen the findings. Based on the present findings, clinical screening for anxiety and depression and estimation of uric acid in the prenatal stage is recommended.

## Conclusion

The present study addressed the contribution of meat consumption patterns in serum uric acid levels and mood disorders in pregnant women in their first trimester. The main findings indicated a significant association of meat consumption, in particular, the consumption of red meat, beef burger and organ meat with increased serum uric acid levels and increased risk of anxiety/depression in pregnant women. The increased serum uric acid levels call for early intervention like a change in diet pattern, such as reasonably consuming lean meat and replacing some portions of meat with fish, also consumption of low-in purine food like eggs, milk and milk products to control uric acid levels and mood disorders. The researchers suggest that maybe the role of increased consumption of meat during pregnancy in increased chances of postpartum depression, which needs further investigation.

### Electronic supplementary material

Below is the link to the electronic supplementary material.

Supplementary Material 1

## Data Availability

The authors affirm that the paper has the information needed to support the study’s findings.

## References

[1] Statista Research Department. Meat consumption Saudi Arabia 2017–2019 [Internet]. 2022. Available from: https://www.statista.com/statistics/1316615/saudi-arabia-meat-consumption/.

[2] Department of Health and Social care. A Quick Guide to the Government’s Healthy Eating Recommendations [Internet]. 2018. Available from: https://assets.publishing.service.gov.uk/government/uploads/system/uploads/attachment_data/file/742746/A_quick_guide_to_govt_healthy_eating_update.pdf.

[3] Intellgence Gmarket. Saudi Arabia Red Meat Market Analysis: Market Segmentation By Meat Type, By Storage, By Distributional Channel & By Region With Forecast 2017–2030 [Internet]. 2020. p. 823. Available from: https://www.goldsteinresearch.com/report/saudi-arabia-red-meat-market.

[4] Global Poultry Trends. Asian Chicken Meat. 2010.

[5] Chen X, Zhao D, Mao X, Xia Y, Baker PN, Zhang H (2016). Maternal dietary patterns and pregnancy outcome. Nutrients.

[6] Skoczyńska M, Chowaniec M, Szymczak A, Langner-Hetmańczuk A, Maciążek-Chyra B, Wiland P (2020). Pathophysiology of hyperuricemia and its clinical significance - a narrative review. Reumatologia.

[7] Choi HK, Liu S, Curhan G (2005). Intake of Purine-Rich Foods, protein, and dairy products and relationship to serum levels of Uric Acid the Third National Health and Nutrition Examination Survey. Arthritis Rheum off J Am Coll Rheumatol.

[8] Kaneko K, Aoyagi Y, Fukuuchi T, Inazawa K, Yamaoka N (2014). Total purine and purine base content of common foodstuffs for facilitating nutritional therapy for gout and hyperuricemia. Biol Pharm Bull.

[9] Kang D-H, Ha S-K (2014). Uric acid puzzle: dual role as anti-oxidantand pro-oxidant. Electrolytes Blood Press E BP.

[10] Kurajoh M, Fukumoto S, Yoshida S, Akari S, Murase T, Nakamura T (2021). Uric acid shown to contribute to increased oxidative stress level independent of xanthine oxidoreductase activity in MedCity21 health examination registry. Sci Rep.

[11] Benn CL, Dua P, Gurrell R, Loudon P, Pike A, Storer RI et al. Physiology of Hyperuricemia and Urate-Lowering Treatments. Front Med [Internet]. 2018;5. Available from: https://www.frontiersin.org/articles/10.3389/fmed.2018.00160. 10.3389/fmed.2018.00160 PMC5990632 29904633

[12] Meng X, Huang X, Deng W, Li J, Li T (2020). Serum uric acid a depression biomarker. PLoS ONE.

[13] Johnson RJ, Kanbay M, Kang D-H, Sánchez-Lozada LG, Feig D. Uric acid: a clinically useful marker to distinguish preeclampsia from gestational hypertension. Vol. 58, Hypertension (Dallas, Tex.: 1979). United States; 2011. p. 548–9. 10.1161/HYPERTENSIONAHA.111.178921 PMC3203211 21876074

[14] Ryu A, Cho NJ, Kim YS, Lee EY (2019). Predictive value of serum uric acid levels for adverse perinatal outcomes in preeclampsia. Med (Baltim).

[15] Starling AP, Sauder KA, Kaar JL, Shapiro AL, Siega-Riz AM, Dabelea D (2017). Maternal dietary patterns during pregnancy are Associated with Newborn Body Composition. J Nutr.

[16] Dunkel Schetter C, Tanner L (2012). Anxiety, depression and stress in pregnancy: implications for mothers, children, research, and practice. Curr Opin Psychiatry.

[17] Nucci D, Fatigoni C, Amerio A, Odone A, Gianfredi V (2020). Red and processed meat consumption and risk of Depression: a systematic review and Meta-analysis. Int J Environ Res Public Health.

[18] Kazemi S, Keshteli AH, Saneei P, Afshar H, Esmaillzadeh A, Adibi P. Red and White Meat Intake in Relation to Mental Disorders in Iranian Adults. Front Nutr [Internet]. 2021;8. Available from: https://www.frontiersin.org/articles/10.3389/fnut.2021.710555. 10.3389/fnut.2021.710555 PMC8353089 34386515

[19] Jacka FN, Pasco JA, Williams LJ, Mann N, Hodge A, Brazionis L (2012). Red meat consumption and mood and anxiety disorders. Psychother Psychosom.

[20] Lu Z, Wang Y, Xun G. Individuals with bipolar disorder have a higher level of uric acid than major depressive disorder: a case–control study. Sci Rep [Internet]. 2021;11(1):18307. 10.1038/s41598-021-97955-4. 10.1038/s41598-021-97955-4 PMC8443646 34526613

[21] Aihemaitijiang S, Zhang Y, Zhang L, Yang J, Ye C, Halimulati M (2020). The Association between Purine-Rich Food Intake. Nutrients.

[22] Tao R, Li H (2015). High serum uric acid level in adolescent depressive patients. J Affect Disord.

[23] Sushil S, Verma N (2010). Questionnaire validation made easy. Eur J Sci Res.

[24] Medicine Iof, Pregnancy. Weight Gain during. Reexamining the guidelines. Washingt DC Natl Acad Sci. 2009.

[25] Terkawi A, Tsang S, AlKahtani G, Al-Mousa S, Al Musaed S, AlZoraigi U et al. Development and validation of Arabic version of the Hospital Anxiety and Depression Scale. Saudi J Anaesth [Internet]. 2017;11(5):11–8. Available from: https://www.saudija.org/article.asp?issn=1658-354X. 10.4103/sja.SJA_43_17 PMC5463562 28616000

[26] Dobersek U, Wy G, Adkins J, Altmeyer S, Lavie CJ, Archer E (2021). Meat and mental health: a systematic review of meat abstention and depression, anxiety, and related phenomena. Crit Rev Food Sci Nutr.

[27] Mofrad MD, Mozaffari H, Sheikhi A, Zamani B, Azadbakht L. The association of red meat consumption and mental health in women: A cross-sectional study. Complement Ther Med. 2021;56(October 2020):102588. 10.1016/j.ctim.2020.102588 33197663

[28] Schmidt JA, Crowe FL, Appleby PN, Key TJ, Travis RC (2013). Serum uric acid concentrations in meat eaters, fish eaters, vegetarians and vegans: a cross-sectional analysis in the EPIC-Oxford cohort. PLoS ONE.

[29] Major TJ, Topless RK, Dalbeth N, Merriman TR. Evaluation of the diet wide contribution to serum urate levels: meta-analysis of population based cohorts. BMJ [Internet]. 2018;363. Available from: https://www.bmj.com/content/363/bmj.k3951. 10.1136/bmj.k3951 PMC6174725 30305269

[30] Wulandari D. The Associations between Age, Stress, High Purine Diet, and Uric Acid Level. Mid-International Conference on Public Health 2018. Indonesia; 2018. p. 257.

[31] Wen S, Cheng M, Wang H, Yue J, Wang H, Li G (2012). Serum uric acid levels and the clinical characteristics of depression. Clin Biochem.

[32] Black CN, Bot M, Scheffer PG, Snieder H, Penninx BW (2018). Uric acid in major depressive and anxiety disorders. J Affect Disord.

[33] Chen J-X, Feng J-H, Zhang L-G, Liu Y, Yang F-D, Wang S-L (2020). Association of serum uric acid levels with suicide risk in female patients with major depressive disorder: a comparative cross-sectional study. BMC Psychiatry.

[34] Ali-Sisto T, Tolmunen T, Velagapudi V, Mäntyselkä P, Lehto SM. Purine Metabolism in Patients with Major Depression: a Follow-up Study. Eur Psychiatry [Internet]. 2015 Mar [cited 2022 Dec 25];30(S1):625. Available from: https://linkinghub.elsevier.com/retrieve/pii/S0924933815319192.

[35] Albert U, De Cori D, Aguglia A, Barbaro F, Bogetto F, Maina G (2015). Increased uric acid levels in bipolar disorder subjects during different phases of illness. J Affect Disord.

[36] Ortiz R, Ulrich H, Zarate CA, Machado-Vieira R (2015). Purinergic system dysfunction in mood disorders: a key target for developing improved therapeutics. Prog Neuro-Psychopharmacology Biol Psychiatry.

[37] Li G, Liu H, Lei L, Zhang X, Gao M, Tu H et al. The Serum Uric Acid Level of Bipolar Depression: A Retrospective Real-World Study. 2021.

[38] Sundström-Poromaa I, Comasco E, Sumner R, Luders E. Progesterone – Friend or foe? Front Neuroendocrinol [Internet]. 2020;59:100856. Available from: https://www.sciencedirect.com/science/article/pii/S0091302220300479. 10.1016/j.yfrne.2020.100856 32730861

[39] Kumar P, Magon N (2012). Hormones in pregnancy. Niger Med J.

[40] Johanson R, Chapman G, Murray D, Johnson I, Cox J. The north staffordshire maternity hospital prospective study of pregnancy-associated depression. J Psychosom Obstet Gynecol [Internet]. 2000;21(2):93–7. 10.3109/01674820009075614. 10.3109/01674820009075614 10994181

[41] Yazici E, Kirkan TS, Aslan PA, Aydin N, Yazici AB (2015). Untreated depression in the first trimester of pregnancy leads to postpartum depression: high rates from a natural follow-up study. Neuropsychiatr Dis Treat.

[42] Luppino FS, de Wit LM, Bouvy PF, Stijnen T, Cuijpers P, Penninx BW (2010). Overweight, obesity, and Depression. Arch Gen Psychiatry.

[43] Pearce M, Garcia L, Abbas A, Strain T, Schuch FB, Golubic R (2022). Association between Physical Activity and Risk of Depression a systematic review and Meta-analysis. JAMA Psychiatry.

[44] Wit L, De, Jelsma JGM, Poppel MNM, Van, Bogaerts A, Simmons D, Desoye G (2015). Physical activity, depressed mood and pregnancy worries in european obese pregnant women: results from the DALI study. BMC Pregnancy Childbirth.

[45] Kołomańska D, Zarawski M, Mazur-Bialy A (2019). Physical activity and depressive Disorders in pregnant women — a systematic review. Med (B Aires).

[46] Murphy SP, Allen LH (2003). Animal Source Foods to improve Micronutrient Nutrition and human function in developing Countries Nutritional Importance of Animal Source Foods 1. J Nutr.

