# Supplementary material for: Implication of red meat consumption habits in serum uric acid levels and mood disorders among first-trimester pregnant women
Source: BMC Nutr. 2023 Sep 29;9:111. doi: 10.1186/s40795-023-00769-y (PMC10541696; doi:10.1186/s40795-023-00769-y)
Supplement: Supplementary file 1 — Supplementary Material 1 [file 40795_2023_769_MOESM1_ESM.pdf]

Meat Frequency Questionnaire

A. How often do you prefer to consume beef, sheep, lamb, veal or goat: grilled, minced, sliced, or shredded? E.g. kabab, kofta, steak

1) Never or less than once a month
2) 1 to 3 times a month
3) 5 to 6 times a week
4) 2 to 4 times a week
5) Once a week
6) More than once daily
7) Once daily

B. How often do you prefer to consume the above mentioned (question " A ") with rice "kabsa"?

1) Never or less than once a month
2) 1 to 3 times a month
3) 5 to 6 times a week
4) 2 to 4 times a week
5) Once a week
6) More than once daily
7) Once daily

C. How often do you prefer to consume the above-mentioned meats (question " A") with broth or stew?

1) Never or less than once a month
2) 1 to 3 times a month
3) 5 to 6 times a week
4) 2 to 4 times a week
5) Once a week
6) More than once daily
7) Once daily

D. How often do you prefer to consume the above-mentioned meats (question " A") with macaroni/ spaghetti /pasta?

1) Never or less than once a month
2) 1 to 3 times a month
3) 5 to 6 times a week
4) 2 to 4 times a week
5) Once a week
6) More than once daily
7) Once daily

E. How often do you prefer to consume meat sandwiches, meat pies, pizza with meat, sausage rolls or any pastry with meat?

1) Never or less than once a month
2) 1 to 3 times a month
3) 5 to 6 times a week
4) 2 to 4 times a week
5) Once a week
6) More than once daily
7) Once daily

F. What is the average consumption of organ meat such as liver, kidneys, heart and lung?

1) Never or less than once a month
2) 1 to 3 times a month
3) 5 to 6 times a week
4) 2 to 4 times a week
5) Once a week
6) More than once daily
7) Once daily

G. What is the average consumption of camel meat?

1) Never or less than once a month
2) 1 to 3 times a month
3) 5 to 6 times a week
4) 2 to 4 times a week
5) Once a week
6) More than once daily
7) Once daily

H. How often do you prefer to consume the above mentioned (question " G ") with rice "kabsa"?

1) Never or less than once a month
2) 1 to 3 times a month
3) 5 to 6 times a week
4) 2 to 4 times a week
5) Once a week
6) More than once daily
7) Once daily

I. How often do you prefer to consume the above-mentioned meats (question " G ") with broth or stew?

1) Never or less than once a month
2) 1 to 3 times a month
3) 5 to 6 times a week
4) 2 to 4 times a week
5) Once a week
6) More than once daily
7) Once daily

J. How often do you prefer to consume sausage meat, pepperoni, and mortadella?

1) Never or less than once a month
2) 1 to 3 times a month
3) 5 to 6 times a week
4) 2 to 4 times a week
5) Once a week
6) More than once daily
7) Once daily

K. Do you eat game meat?

1) No (GO TO QUESTION "O")
2) Yes (ANSWER the following QUESTION from L to N)

L. If the answer to the previous question is yes, which of the following is consumed?
1) rabbit
2) gazelle/deer
3) birds
4) jerboa
5) ostrich
6) Dabb lizards/or Spiny-tailed lizards/or Mastigures
7) Other: please specify

M. According to the above choice, what is the average consumption?
1) Never or less than once a month
2) 1 to 3 times a month
3) 5 to 6 times a week
4) 2 to 4 times a week
5) Once a week
6) More than once daily
7) Once daily

N. What is the average consumption of birds: pigeons, quail…etc?
1) Never or less than once a month
2) 1 to 3 times a month
3) 5 to 6 times a week
4) 2 to 4 times a week
5) Once a week
6) More than once daily
7) Once daily

O. How often do you prefer to consume beef burgers?
1) Never or less than once a month
2) 1 to 3 times a month
3) 5 to 6 times a week
4) 2 to 4 times a week
5) Once a week
6) More than once daily
7) Once daily

P. How often do you prefer to consume chicken burgers?
1) Never or less than once a month
2) 1 to 3 times a month
3) 5 to 6 times a week
4) 2 to 4 times a week
5) Once a week
6) More than once daily
7) Once daily

Q. How often do you prefer to consume chicken and other poultry, for example: turkey, duck, geese, etc.?
1) Never or less than once a month
2) 1 to 3 times a month
3) 5 to 6 times a week
4) 2 to 4 times a week
5) Once a week

6) More than once daily
7) Once daily

R. How often do you prefer to consume poultry mentioned in the (question “ Q”) : as for example, pie, sandwich, pastries, rolls?

1) Never or less than once a month
2) 1 to 3 times a month
3) 5 to 6 times a week
4) 2 to 4 times a week
5) Once a week
6) More than once daily
7) Once daily

S. How often do you prefer to consume sausage, pepperoni, and mortadella according to (question “ Q ”)?

1) Never or less than once a month
2) 1 to 3 times a month
3) 5 to 6 times a week
4) 2 to 4 times a week
5) Once a week
6) More than once daily
7) Once daily

T. How often do you prefer to consume fresh or frozen white fish, for example cod, grouper, Coral trout, Parrot Fish, haddock..etc?

1) Never or less than once a month
2) 1 to 3 times a month
3) 5 to 6 times a week
4) 2 to 4 times a week
5) Once a week
6) More than once daily
7) Once daily

U. How often do you prefer to consume fresh or canned oily fish, for example Mackerel, tuna, salmon, sardines and herring, etc?

1) Never or less than once a month
2) 1 to 3 times a month
3) 5 to 6 times a week
4) 2 to 4 times a week
5) Once a week
6) More than once daily
7) Once daily

V. How often do you prefer to consume fried fish, finger fish, fish pie, etc?

1) Never or less than once a month
2) 1 to 3 times a month
3) 5 to 6 times a week
4) 2 to 4 times a week
5) Once a week
6) More than once daily
7) Once daily

W. How often do you prefer to consume seafood, eg crab, shrimp, mussels, lobster?

1) Never or less than once a month
2) 1 to 3 times a month
3) 5 to 6 times a week
4) 2 to 4 times a week
5) Once a week
6) More than once daily
7) Once daily

***In general*, what is the preferred way to cook the following meat?**

X. Red meat:

1) Roasting: meat is cooked in an oven. The meat is not covered and no water is added.
2) Broiling or Grilling
3) Pan-broiling: it is cooked in an uncovered pan over direct heat. Fat that cooks out of the meat is drained off.
4) Pan-frying: meat is cooked in a small amount of fat.
5) Stir-frying: it is stirred almost continuously. Cooking is done at high heat, using small or thin pieces of meat.
6) Deep-fat frying
7) Braising: it is cooked in steam trapped and held in a covered container or foil wrap
8) Cooking in liquid: by using the three ways to cook in liquid are simmering, stewing and poaching.

Y. White meat;

1) Roasting: meat is cooked in an oven. The meat is not covered and no water is added.
2) Broiling or Grilling
3) Pan-broiling: it is cooked in an uncovered pan over direct heat. Fat that cooks out of the meat is drained off.
4) Pan-frying: meat is cooked in a small amount of fat.
5) Stir-frying: it is stirred almost continuously. Cooking is done at high heat, using small or thin pieces of meat.
6) Deep-fat frying
7) Braising: it is cooked in steam trapped and held in a covered container or foil wrap
8) Cooking in liquid: by using the three ways to cook in liquid are simmering, stewing and poaching.

Z. Fish

1) Roasting: meat is cooked in an oven. The meat is not covered and no water is added.
2) Broiling or Grilling
3) Pan-broiling: it is cooked in an uncovered pan over direct heat. Fat that cooks out of the meat is drained off.
4) Pan-frying: meat is cooked in a small amount of fat.
5) Stir-frying: it is stirred almost continuously. Cooking is done at high heat, using small or thin pieces of meat.
6) Deep-fat frying
7) Braising: it is cooked in steam trapped and held in a covered container or foil wrap
8) Cooking in liquid: by using the three ways to cook in liquid are simmering, stewing and poaching.

AA. Do you eat any other kinds of meat besides those listed above? or employ any other cooking technique not covered above?

1) No
2) Yes

If yes, please specify

BB. Other information that you would like to add

Thank you
